# A LASSO penalized regression approach for genome-wide association analyses using related individuals: application to the Genetic Analysis Workshop 19 simulated data

**DOI:** 10.1186/s12919-016-0034-9

**Published:** 2016-10-18

**Authors:** Charalampos Papachristou, Carole Ober, Mark Abney

**Affiliations:** 1 Department of Mathematics, Physics, and Statistics, University of the Sciences, 600 S. 43rd Street, Philadelphia, PA 19104 USA; 2 Department of Mathematics, Rowan University, 201 Mullica Hill Road, Glassboro, NJ 08028 USA; 3 Department of Human Genetics, University of Chicago, 920 E. 58th Street, CLSC 4th floor, Chicago, IL 60637 USA

## Abstract

We propose a novel LASSO (least absolute shrinkage and selection operator) penalized regression method used to analyze samples consisting of (potentially) related individuals. Developed in the context of linear mixed models, our method models the relatedness of individuals in the sample through a random effect whose covariance structure is a linear function of known matrices with elements combinations of the condensed coefficients of identity between the individuals in the sample. We implement our method to analyze the simulated family data provided by the 19th Genetic Analysis Workshop in an effort to identify loci regulating the simulated trait of systolic blood pressure. The analyses were performed with full knowledge of the simulation model. Our findings demonstrate that we can significantly reduce the rate of false positive signals by incorporating the relatedness of the study participants.

## Background

Current association methods for quantitative phenotypes are mostly designed for use with unrelated individuals. However, often studies include information on families or related individuals. Also, many times samples assumed to include only independent individuals may suffer from underlying cryptic relatedness among the participants. Using related individuals in statistical analyses has many advantages, such as increased ability for quality control and robustness of the results with respect to population stratification. Ignoring relatedness between study participants can have significant impact on the study results and increase false positive rates [1]. Linear mixed models (LMMs) have been successfully used in genome-wide association studies (GWAS) to model effects shared by groups of individuals in the sample such as common genetic background or population stratification [1–4].

Because of the computational intensity involved in the estimation of the parameters of the LMM, most methods perform single marker analysis [5]. As GWAS typically include a very large number of single-nucleotide polymorphisms (SNPs), it is imperative to control for type I error. Typically, this is achieved through the use of some multiplicity adjustment method such as Bonferonni’s. Lately, least absolute shrinkage and selection operator (LASSO) regression [6] has attracted attention as an alternative tool for selecting the most promising SNPs in GWAS [5, 7]. LASSO regression has the advantage that, by modelling multiple SNPs simultaneously, it can distinguish trait contributing loci from loci that are in high linkage disequilibrium with those loci. Currently, all LASSO methods used in GWAS assume that the sample members are unrelated to each other. However, this may not be true as often genetic studies recruit multiple members of families. As such, researchers usually restrict analyses to seemingly unrelated members of the samples. Alternatively, one can first run analyses based on LMM to remove the effects of relatedness among individuals and then use the residuals of these analyses to fit the LASSO models [7]. These approaches, however, can lead to loss of power as they make suboptimal use of the data. Thus, a LASSO version that accounts for potential relatedness between study subjects is needed.

We have developed a new association method that combines LMM and LASSO to capitalize on the benefits of both approaches. Our method handles multiple markers and models (potential) common genetic background among individuals through the use of a random polygenic effect. It employs a LASSO algorithm [8] to estimate the model parameters and ultimately select loci potentially important in the regulation of a quantitative phenotype. We analyze all 200 replicates of the simulated family data available by the 19^th^ Genetic Analysis Workshop (GAW19) [9] to study the properties of the method with regards to its ability to yield true signals while maintaining low false positives.

## Methods

Consider a sample of *n* (potentially) related individuals. Let *y* = (*y*_1_, …, *y*_*n*_) be the vector of the phenotypic values of the quantitative trait of interest. We assume that for each person *i* the value of his/her phenotype is determined (potentially) by a number, say *S*, of (diallelic) loci/SNPs, hereafter referred to as quantitative trait loci (QTLs). Furthermore, the trait is also influenced (potentially) by *K* known covariates *X*_*i*_, *i = 1,…, K*, (eg, age). In addition, there is some environmental effect or residual, denoted as *e*_*i*_. Finally, we suppose the phenotype is affected by a number of other loci whose collective (random) polygenic effect on the trait is denoted as *u*_*i*_. Assuming additivity across all effects, the overall phenotypic value of a person is

1 $$ {y}_i=\beta {X}_i+\gamma {Z}_i+{u}_i+{e}_i $$

where *β =* (*β*_1_, …, *β*_*K*_) is a vector of unknown coefficients for the covariate fixed effects, *γ =* (*γ*_1_, …, *γ*_*S*_) is a vector of unknown coefficients of the fixed effect of the SNPs, *Z*_*i*_ *=* (*z*_1_, …, *z*_*L*_) a vector of 0, 1, or 2 s, denoting the number of copies of the disease (minor) allele carried by the individual at each of the loci. Note that, if a SNP does not contribute to the trait levels, we expect its corresponding coefficient *γ*_*s*_ to be equal to zero. We assume that the vector of random polygenic effects *u* = (*u*_1_, …, *u*_*n*_) follows a multivariate normal distribution with mean 0 and covariance matrix *Ω*, while the errors *e*_*i*_s are independent and identically distributed coming from a normal distribution with mean 0 and variance. Finally, the two random effects, *u*_*i*_ and *e*_*i*_, are considered independent of each other and the covariates. Under these assumptions, the joint distribution of the *y*_*i*_’s is a multivariate normal distribution with mean equal to *βX* + *γZ* and variance covariance matrix

2 $$ {V}_y=\Omega +{\sigma}_e^2I $$

where *X* ^*T*^ = (*X*_1_, …, *X*_*n*_), *Z* ^*T*^ = (*Z*_1_, …, *Z*_*n*_), and *I* is the *n* × *n* identity matrix. If there is minimal or no inbreeding among the individuals in the sample, then the matrix *Ω* is approximately equal to

3 $$ \Omega =2\varPhi {\sigma}_{ap}^2+\varDelta {\sigma}_{dp}^2 $$

where *Φ* and *Δ* are known matrices whose elements are functions of the condensed coefficients of identity between the individuals in the sample, and *σ*_*ap*_^2^ and *σ*_*dp*_^2^ are the additive and dominance polygenic variance of the trait [4, 10].

Estimation of the model parameters is done by maximizing the corresponding multivariate normal likelihood. The fitting of the model, though, is computationally intense and plagued by many issues because of the high dimensionality of the parameter space and the small available sample size. However, as most *γ*s are expected to be negligible, a LASSO method may be useful as it generally forces most of the parameters in the model to shrink toward zero with only a small number of the parameters remaining significantly different from zero. In our case, the penalized likelihood function that needs to be maximized to estimate the necessary parameters is given by the following equation:

4 $$ {Q}_{\lambda}\left(\beta, \gamma, \theta \right)\propto \log \left|V\left(\theta \right)\right|+{\left(y-X\beta -Z\gamma \right)}^T{V}^{-1}\left(\theta \right)\left(y-X\beta -Z\gamma \right)+\lambda {\displaystyle {\sum}_{s=1}^S}\left|{\beta}_s\right| $$

where *θ* = (*σ*_*a*_^2^, *σ*_*d*_^2^, *σ*_*e*_^2^) and *λ* is a nonnegative penalty parameter. Maximization of the above function can be achieved using an algorithm similar to that described by Schelldorfer et al. [8].

## Results

For our analyses, we used the family-simulated data sets from GAW19 [9] to uncover QTLs regulating the simulated quantitative phenotype systolic blood pressure (SBP). The simulation model included 983 functional QTLs affecting the phenotype scattered over the 11 chromosomes for which genotypes were provided. To study the properties of the proposed method in terms of the true and false discovery rates (denoted as TDR and FDR, respectively), we analyzed all 200 replicates provided, using the 845 individuals in 20 extended pedigrees for whom there was information on both the genotypes and phenotypes. Note that the genotypes of all the individuals were identical in all replicates. The only information varying from replicate to replicate was the simulated phenotypes.

We used the SBP at the last known appointment as the phenotype of interest and the imputed genotypes provided as those were used in the generation of the simulated phenotypes. Because of the large number of SNPs available, greater than 8,000,000, we preprocessed the genotypes to reduce the number of SNPs used in the analyses to reduce the computational burden. We excluded SNPs with minor allele count less than 10 copies in the entire data set (roughly minor allele frequency <0.01) to avoid potential problems with unstable estimates of the model parameters in equation (1). We spaced the markers so that the minimum distance between consecutive SNPs was at least 0.5 kbp. We did not consider filtering criteria that were based on the genotyping rate, call rate, or Hardy-Weinberg equilibrium as the data had already been cleaned with respect to those. The preprocessing of the genotypes resulted in a genetic map consisting of 952,366 SNPs. Not surprisingly, of the original 983 simulated QTLs, only 115 remained in the reduced map as the vast majority of the QTLs under the simulation model had rare minor alleles.

The number of SNPs on the map was prohibitively large for directly implementing our approach. So, we implemented a 2-step scheme to reduce the number of SNPs used when fitting the penalized model. Two-step methods are routinely employed when the computational intensity of a method prohibits direct use of the method on a GWAS [4]. In phase I, we performed a genome-wide scan using the association test for independent individuals as implemented in PLINK [11] to identify potentially “interesting” SNPs to be followed up in phase II. We essentially used the same data on both phases of the analysis so results from the 2 analyses are dependent. However, the lack of independence does not pose a major issue in the validity of the results as the purpose of the 2-stage analysis is only to reduce the computational intensity and not for cross-validation. The phase I analysis was based only on data from the 295 unrelated individuals in the sample, while phase II used all 845 related individuals. In phase II, all SNPs that exceeded the set *p* value threshold (10E-04 or 10E-05) were used to fit the LASSO model. In all analyses in phase I or II, phenotypes were adjusted for gender, age, smoking status, and blood pressure medication.

The results from the analyses of all replicates are summarized in Table 1. The threshold for declaring a SNP “significant” on phase I was set to a *p* <1E-05. A SNP was called as “significant” if its *p* value was less than the given threshold (for the PLINK analysis) or if its coefficient was different from zero in the optimal LASSO model. We present the results for 3 LASSO analyses. The first, Add, represents an analysis in which the random polygenic effect was assumed to consist of only additive genetic variance, that is, the covariance structure was assumed to be Ω = 2*Φσ*_*a*_^2^. The second model, Add-Dom, assumed that the trait had also a dominance effect and thus the matrix *Ω* was set to have the form Ω = 2*Φσ*_*a*_^2^ + *Δσ*_*d*_^2^. Finally, for comparison, we also implemented the standard LASSO method that assumes that all individuals in the study are unrelated, denoted as Ind. The standard LASSO approach is a subcase of our approach where matrix *Ω* is the 0 matrix.

**Table 1:** Results of the analyses of the 200 replicates of the simulated data for SBP

Method^a^	IC^b^	Significant SNPs^c^		QTLs discovered^d^			SNPs within 2 kbp of QTL^e^		
		Mean^f^	SD^f^	TDR^g^	Mean^f^	SD^f^	TDR^g^	Mean^f^	SD^f^
PLINK	–	137.1	98.9	0.020	0.02	0.14	0.240	0.23	0.60
Add	AIC	14.4	5.3	0.000	0.00	0.00	0.010	0.01	0.10
	BIC	7.0	3.7	0.000	0.00	0.00	0.010	0.01	0.10
Add-Dom	AIC	14.4	5.3	0.000	0.00	0.00	0.010	0.01	0.10
	BIC	7.1	3.7	0.000	0.00	0.00	0.010	0.01	0.10
Ind	AIC	22.1	9.1	0.000	0.00	0.00	0.020	0.02	0.14
	BIC	15.0	6.2	0.000	0.00	0.00	0.010	0.01	0.10

^a^Method used/covariance structure of random polygenic effect (for LASSO): *PLINK* PLINK analysis results, *Add* assuming only additive genetic variance, *Add-Dom* assuming both additive and dominance genetic effect, *Ind* assuming members independent

^b^Information criterion used to select best model: *AIC* Akaike, *BIC* Bayesian

^c^Number of SNPs with a *p* value <1E-05 (PLINK) or nonzero coefficient on the optimal LASSO model

^d^Number of actual simulated QTLs with a *p* value <1E-05 (PLINK) or nonzero coefficient on the optimal LASSO model

^e^Number of SNPs with a *p* value <1E-05 (PLINK) or nonzero coefficient on the optimal LASSO model located within 2 kbps from a QTL

^f^Mean and standard deviation of the number of SNPs over the 200 replicates

^g^Proportion of replicates with *at least* 1 SNP/QTL with a *p* value <1E-05 (PLINK)/nonzero coefficient on the optimal LASSO model

The first group of columns in the table, “Significant SNPs,” summarizes the number of SNPs declared “significant” by each method. The second section, “QTLs discovered,” summarizes the number of QTLs declared “significant” and is used to gauge the TDR of the method. This column uses a very strict definition of what constitutes “true” hit. A more relaxed rule for what constitutes a true signal considered as a true hit any SNP that resided within (an arbitrary) 2 kbp from any QTL. There were 2248 SNPs on the map that were within 2 kbp from a simulated QTL. The results using this definition are summarized in the “SNPs within 2 kbp from QTL” section of the table.

From Table 1, we can see that, even though PLINK identified, on average, 137.1 SNPs as significant, in only 2 % of the replicates did these SNPs include any simulated QTL, while approximately 24 % of the replicates included at least 1 significant SNP within 2 kbp of a QTL. This result, along with the fact that of the 115 QTLs remaining after genotype cleaning PLINK was able to identify approximately 0.02 QTLs per replicate, suggests a poor performance of the method in phase I. This performance can be partially explained by the low contribution of each QTL on the phenotype and the small sample size, which resulted in low power to detect these QTLs.

The results from the LASSO analyses show that modeling the relatedness of the individuals in the sample pays off, regardless of whether we did (Add-Dom) or did not (Add) model the dominance effect. The LASSO method for related individuals, on average, reduced the number of SNPs with nonzero coefficients in the final model relatively to the corresponding number from the standard LASSO method (Ind). For example, when the Bayesian information criterion (BIC) was used to select the best model, the Ind model resulted in 15 significant SNPs, whereas both the Add and the Add-Dom models included, on average, approximately 7 “significant” SNPs,, marking a reduction of approximately 53 % in the number of “significant” SNPs. For this particular data, it appears that using the more complex modeling of the covariance structure of the random effect had no significant effect on the performance of the method, as the results from both the additive (Add) and the additive-dominance (Add-Dom) models were almost identical. This was expected as the particular phenotype (SBP) was generated using a model with only additive effects. The choice of the information criterion was also not surprising. BIC being stricter resulted in models that, on average, included fewer SNPs with nonzero coefficients, 7.1 from 137.1, than when Akaike information criterion (AIC) was used. The actual TDR of the method, though, appears very low (approximately 1 %). This is not surprising as the TDR of the method was affected by the low discovery rate of the PLINK analysis.

To further study the TDR of the LASSO method, we reanalyzed the data using a less-strict threshold, *p* <1E-04, for declaring significant SNPs with the PLINK analysis in hopes of increasing the number of actual simulated QTLs “identified” in the phase I analyses. For the LASSO model, we opted not to fit the Add-Dom model as it significantly increases the analysis time and, based on the analysis above, we did not anticipate the results of the 2 models to significantly differ. Table 2 presents the results. Even though the new threshold in the GWAS analysis resulted in an average of approximately 695.8 significant SNPs, it did not increase the number of true hits. The PLINK analysis uncovered at least 1 QTL in approximately 74 % of the replicates, with an average of 1.9 SNPs within 2 kbp from a simulated QTL. Consequently, the TDR of the LASSO methods was low, less than 20 %, with the models resulting from the BIC criterion having an even lower TDR. In contrast, the LASSO model with the additive component was able to significantly reduce the number of SNPs with nonzero coefficients in the optimal model from approximately 695.8 SNPs to only 14.1, marking a 3-fold decrease from the corresponding number from the independent LASSO (41.2 SNPs on average).

**Table 2:** Results of the analyses using *p* value threshold of 1E-04 for the PLINK analyses^a^

Method	IC	Significant SNPs		QTLs Discovered			SNPs within 2 kbp of QTL		
		Mean	SD	TDR	Mean	SD	TDR	Mean	SD
PLINK	–	695.8	287.7	0.135	0.13	0.36	0.735	1.89	1.87
Add	AIC	37.4	17.1	0.005	0.01	0.07	0.120	0.12	0.33
	BIC	14.1	5.8	0.000	0.00	0.00	0.015	0.02	0.13
Ind	AIC	63.84	21.0	0.005	0.01	0.07	0.200	0.20	0.47
	BIC	41.2	10.6	0.005	0.01	0.07	0.130	0.13	0.38

^a^For definitions of the abbreviations refer to the footnotes to Table 1

In an effort to increase the number of true QTLs identified, we repeated the analyses, using the genome-wide efficient mixed-model analysis (GEMMA) software [3] in phase I to perform the GWAS. GEMMA performs single-marker analyses for multiple related individuals by modeling relatedness using a random polygenic effect whose covariance structure has the same form as the one we used in our LASSO method. Because the method can handle families of arbitrary size and structure, we used all available data for both stages of the analyses (I and II). Table 3 summarizes the results for a *p* value threshold of 1E-04 for the GEMMA analyses.

**Table 3:** Results of the analyses using *p* value threshold of 1E-04 for the GEMMA analyses^a^

Method	IC	Significant SNPs		QTLs Discovered			SNPs within 2 kbp of QTL		
		Mean	SD	TDR	Mean	SD	TDR	Mean	SD
GEMMA	–	583.8	195.9	0.600	0.7	0.7	0.800	4.1	3.3
Add	AIC	84.4	15.1	0.100	0.1	0.2	0.300	0.4	0.7
	BIC	25.3	25.7	0.000	0.0	0.2	0.200	0.2	0.4
Ind	AIC	105.2	16.2	0.000	0.0	0.2	0.300	0.4	0.6
	BIC	80.6	18.0	0.000	0.0	0.2	0.300	0.3	0.6

^a^For definitions of the abbreviations refer to the footnotes on Table 1

Table 3 shows that use of all available data to identify potential QTLs in phase I of the analysis slightly increased the power of the method to identify at least 1 true simulated QTL to 80 % from 73.5 % when PLINK was used. Furthermore, GEMMA slightly increased the number of SNPs within 2 kbp of a true simulated gene from 1.89 using PLINK to approximately 4.1, on average, even though fewer SNPs advanced to phase II (583.8 vs. 695.8, on average). Similarly, the 2-step method using GEMMA resulted in a small increase in power to identify at least 1 SNP within 2 kbp of a QTL to 20 % from 12 % when PLINK was used in phase I. However, the cost of the increase in the power was a significant increase in the number of SNPs with nonzero coefficients in the final model. The best model based on the BIC criterion on average resulted in 25.3 “significant” SNPs, up from 14.1 when PLINK was used in phase I. This behavior is not surprising. Our LASSO method and GEMMA model the quantitative phenotype in the same way; consequently, LASSO tends to overfit the data, resulting in a larger number of SNPs with nonzero coefficients in the final model.

## Discussion

We have presented a 2-stage approach that uses a LASSO method for analyzing quantitative phenotypes of (potentially) related individuals. We first perform a GWAS to uncover SNPs potentially associated with the regulation of the phenotype. Next, a multivariate LASSO approach that accounts for relevant covariates and relatedness among the study participants is used to further weed out additional SNPs. The method models relatedness of individuals in the sample through inclusion of a random polygenic effect whose covariance structure reflects relatedness of the individuals. We used the GAW19 simulated family data to study the properties of the method and to compare its performance to the standard LASSO for unrelated individuals.

In application to the GAW19 simulated data, our results indicate that incorporating the relatedness of individuals in the LASSO model can help significantly reduce the number of non–trait-regulating SNPs falsely inferred as associated with the trait relatively to the standard LASSO approach, which assumes unrelated individuals. Furthermore, for these particular data, it appears that including a dominance genetic effect in the model has no significant effect other than to increase the computational intensity of the method. This was expected as the particular data were generated without dominance effects.

## Conclusions

The power of the LASSO method appears to be small, mainly as a result of the inability of the initial GWAS scan to identify QTLs to take forward for further analysis. In an attempt to increase the number of QTLs selected for the phase II analysis, we explored 2 methods for the GWAS: (a) using PLINK on only the founders of the pedigrees, and (b) using GEMMA to allow for related individuals and use of all study participants. Unfortunately, even though the GEMMA analysis yielded a slightly larger number of true signals, neither of the 2 methods was able to advance to the next stage a significant number of true QTLs. This behavior could potentially be a result of the low contribution of the simulated QTLs to the phenotype. Perhaps using other methods in stage I, such as the transmission disequilibrium test or family-based association test, on an appropriate subset of the data, may increase the number of QTLs that are carried forward, thereby potentially increasing the TDR of the method.
